# Development of a core outcome set for clinical trials of interventions to improve sleep in people with cognitive impairment‐the Sleep in Cognitive Impairment Core Outcome Set (SCICOS)

**DOI:** 10.1002/alz.70890

**Published:** 2025-11-06

**Authors:** Patrick Crowley, Alasdair L. Henry, Evelyn Flanagan, Inga Antonsdottir, Alison Bentley, Jonathan Blackman, Donald L. Bliwise, Omonigho M. Bubu, Daniel J. Buysse, Einstein F. Camargos, Erin Cassidy‐Eagle, Kimberly Cote, Elizabeth Coulthard, Angela L. D'Rozario, Colin A. Espie, Ryan S. Falck, Victoria G. Gabb, Allison G. Harvey, Nant Thin Thin Hmwe, Camilla M. Hoyos, Lucy Jobbins, Seán Kennelly, Brianne A. Kent, Sascha Köpke, Andrew Krystal, Iracema Leroi, Claudio Liguori, Yen Ying Lim, Rebecca Lorenz, Brendan P. Lucey, Bryce Mander, Margaret Moline, Sharon L. Naismith, Adesola Ogunniyi, Penny Rapaport, Charles F. Reynolds, Kathy Richards, Catherine F. Siengsukon, Shireen Sindi, Clifford M. Singer, Anna Wirz‐Justice, Kristine Yaffe, Rónán O'Caoimh

**Affiliations:** ^1^ Department of Geriatric Medicine Mercy University Hospital Cork Ireland; ^2^ Health Research Board Clinical Research Facility‐Cork University College Cork Mercy University Hospital Cork Ireland; ^3^ Big Health Ltd London UK; ^4^ Johns Hopkins School of Nursing, Department of Psychiatry and Behavioral Sciences Richman Family Precision Medicine Center of Excellence in Alzheimer's Disease, Johns Hopkins Bayview Johns Hopkins Medicine Baltimore Maryland USA; ^5^ Restonic Ezintsha Sleep Clinic Johannesburg South Africa; ^6^ Bristol Medical School University of Bristol Bristol UK; ^7^ Department of Neurology, Emory University School of Medicine, Department of Adult and Elder Health, Nell Hodgson School of Nursing Emory University Atlanta Georgia USA; ^8^ Departments of Psychiatry, Neurology, and Population Health at Grossman School of Medicine New York University New York USA; ^9^ Department of Psychiatry University of Pittsburgh School of Medicine Pittsburgh Pennsylvania USA; ^10^ Postgraduate Program in Medical Sciences University of Brasília Brasília Brazil; ^11^ Department of Psychiatry and Behavioral Sciences Stanford University School of Medicine Palo Alto California USA; ^12^ Department of Psychology Brock University St. Catharines Ontario Canada; ^13^ School of Psychological Sciences Macquarie University Sydney New South Wales Australia; ^14^ Nuffield Department of Clinical Neurosciences Oxford University Oxford UK; ^15^ School of Biomedical Engineering University of British Columbia Vancouver British Columbia Canada; ^16^ Department of Psychology University of California Berkeley Berkeley California USA; ^17^ Department of Nursing Science Faculty of Medicine Universiti Malaya Kuala Lumpur Malaysia; ^18^ Centre for Sleep and Chronobiology Woolcock Institute of Medical Research Macquarie University Sydney New South Wales Australia; ^19^ Department of Medical Gerontology Trinity College Dublin Dublin Ireland; ^20^ Department of Psychology Simon Fraser University Burnaby British Columbia Canada; ^21^ Institute of Nursing Science, Faculty of Medicine and University Hospital Cologne University of Cologne Cologne Germany; ^22^ Departments of Psychiatry and Neurology University of California San Francisco San Francisco California USA; ^23^ School of Medicine and Global Brain Health Institute Trinity College Dublin Dublin Ireland; ^24^ Department of Systems Medicine, and Neurology Unit University Hospital of Rome Tor Vergata Rome Italy; ^25^ Turner Institute for Brain and Mental Health Monash University Clayton Victoria Australia; ^26^ College of Nursing The Ohio State University Columbus Ohio USA; ^27^ Department of Neurology Washington University School of Medicine St. Louis Missouri USA; ^28^ Department of Psychiatry and Human Behavior University of California Irvine Irvine California USA; ^29^ Eisai Inc. Nutley New Jersey USA; ^30^ School of Psychology Faculty of Science The University of Sydney Camperdown New South Wales Australia; ^31^ Department of Medicine University of Ibadan Ibadan Nigeria; ^32^ Division of Psychiatry University College London London UK; ^33^ School of Medicine University of Pittsburgh Pittsburgh Pennsylvania USA; ^34^ School of Nursing University of Texas Austin Texas USA; ^35^ Department of Physical Therapy Rehabilitation Science and Athletic Training University of Kansas Medical Center Kansas City Kansas USA; ^36^ Division of Clinical Geriatrics Center for Alzheimer Research Karolinska Institutet and Karolinska University Hospital Stockholm Sweden; ^37^ Ageing Epidemiology Research Unit (AGE) School of Public Health Faculty of Medicine Imperial College London London UK; ^38^ Memory and Aging Program Northern Light Acadia Hospital UMaine Institute of Medicine University of Maine Bangor Maine USA; ^39^ Centre for Chronobiology Psychiatric Clinic University of Basel Basel Switzerland; ^40^ Departments of Psychiatry Neurology and Epidemiology University of California San Francisco San Francisco California USA

**Keywords:** Alzheimer's disease, core outcome set, dementia, mild cognitive impairment, sleep

## Abstract

**INTRODUCTION:**

Sleep disturbances are common in older people with cognitive impairment, potentially contributing to negative outcomes. A core outcome set (COS) is required to reduce heterogeneity in clinical trials and promote the development of high‐quality evidence to support clinical management.

**METHODS:**

A multi‐stage mixed methods study was conducted in accordance with The Core Outcome Set Standards for Development.

**RESULTS:**

A systematic review identified 287 sleep outcomes from previous clinical trials. Qualitative interviews ensured the COS was informed by what matters most to people with cognitive impairment and their caregivers. A modified Delphi process identified nine outcomes for the COS: total sleep time, sleep onset latency, wakefulness after sleep onset, number of night‐time awakenings, sleep efficiency, and measures of sleep quality, daytime sleepiness, cognition, and mood.

**DISCUSSION:**

This COS will support researchers to produce more reliable and coherent trial data to guide the management of sleep disturbances in people with neurodegenerative cognitive impairment.

**Highlights:**

- Evidence is lacking regarding the treatment of sleep disturbances in people with cognitive impairment.
- Heterogeneity of reported outcomes in clinical trials limits data synthesis.
- A qualitative analysis established what matters most to people with cognitive impairment and their caregivers when determining treatment effectiveness.
- A Delphi panel of experts agreed upon a core outcome set.
- This core outcome set will improve the reliability and comparability of data from future trials.

## 1 BACKGROUND

Dementia is a major neurocognitive disorder characterized by progressive cognitive impairment caused by one or more pathological processes, distinct from normal physiological aging, leading to impairment of everyday functioning.^1^ There are approximately 55 million people living with dementia worldwide and, with a rapidly growing and aging population, the global prevalence of dementia is expected to increase to 139 million by 2050.^2^ Despite the recent emergence of disease‐modifying therapies,^3^, ^4^ there is no curative treatment. Clinical management, therefore, remains largely focused on addressing modifiable risk factors to prevent the development and progression of dementia, and on reducing the burden of symptoms in more advanced cases.

There are well established yet multifaceted and, in some aspects, imprecisely defined, associations between sleep disturbances and cognitive impairment across the clinical continuum.^5^, ^6^, ^7^ Mild cognitive impairment (MCI) describes a prodromal stage of dementia when, despite objective evidence of cognitive impairment, independent functioning is preserved.^8^ Studies show that, compared to healthy age‐matched controls, people with MCI experience reduced total sleep time, sleep efficiency, and duration of rapid eye movement (REM) sleep, while sleep onset latency (SoL), wakefulness after sleep onset, and duration of non‐REM (NREM) stage one sleep are increased.^9^, ^10^ There is emerging evidence that the causal relationship between sleep disturbances and dementia may be bi‐directional.^11^, ^12^ A recent meta‐analysis of observational studies calculated a population attributable risk percentage, suggesting that 15% of Alzheimer's disease may be attributable to sleep disturbances.^5^ Despite the recent dementia prevention, intervention and care 2024 report of the Lancet Commission, concluding that further research is needed to clarify the effect of sleep disturbances on cognitive decline,^13^ interest is growing in sleep as a potential modifiable risk factor for dementia.^12^, ^14^ The European Academy of Neurology Brain Health Strategy, for example, has included sleep as a determinant of brain health.^15^

Sleep disturbances tend to worsen as cognition declines,^16^ leading to deteriorating cognitive outcomes^17^ and quality of life.^18^ Furthermore, relationships with caregivers can become strained,^19^ sometimes resulting in sub‐optimal care^20^ and early institutionalization.^21^ Sleep disturbances are therefore an important treatment target throughout the clinical course of dementia, to potentially slow the progression of early‐stage disease and to reduce the burden of symptoms in more advanced cases. Despite this, there is a paucity of evidence supporting the treatment of sleep disturbances in this population.^22^ Systematic reviews of interventions to improve sleep in people with cognitive impairment have been limited by small sample sizes with wide heterogeneity, both in the manner in which sleep is measured and in the outcomes reported, limiting data synthesis.^23^, ^24^

A recent scoping review of the measurement of sleep in people with MCI and mild dementia found that, after synonymous terms had been merged, 165 different sleep outcomes were reported across all included studies.^25^ Of these, 62 (37.6%) were reported in only one of the included studies. There was also notable variability in the methods used to measure sleep. Of the 188 studies included in the review, 131 used subjective measures of sleep such as questionnaires or sleep diaries. Objective measures were used less frequently, with 88 of the included studies using polysomnography and 37 using actigraphy. This heterogeneity, both in the methods used to measure sleep and in the outcomes reported, limits the comparability of data, precludes data synthesis, and impedes the development of a robust evidence base to guide clinical practice.

A core outcome set (COS) is therefore required to improve the coherence and reliability of data from future clinical trials in this field. A COS is an agreed standardized set of outcomes that should be measured and reported, as a minimum, in all clinical trials in specific areas of health or health care.^26^ This study involved key stakeholders in the development of a COS for clinical trials of interventions to improve sleep in people with neurodegenerative cognitive impairment, across all stages from MCI to severe dementia—the Sleep in Cognitive Impairment Core Outcome Set (SCICOS).

**RESEARCH IN CONTEXT**

1. **Systematic review**: A systematic review of Medline (Ovid), CINAHL, PsycINFO, and the Cochrane CENTRAL database revealed that 287 discrete sleep outcomes were used in previous clinical trials of interventions to improve sleep in people with mild cognitive impairment and dementia. Of these, 205 were used in only one of the studies included in the review. This heterogeneity in reported outcomes limits the comparability of data, precluding data synthesis and impeding the development of a robust evidence base to guide clinical management.
2. **Interpretation**: After consultation with people living with cognitive impairment and their caregivers to understand what matters most to them, a modified Delphi process involving experts in sleep and cognition identified a core outcome set that should be reported in future clinical trials.
3. **Future directions**: This core outcome set will help ensure that future clinical trials produce more reliable and coherent data to guide management of sleep disturbances in people with mild cognitive impairment and dementia.

## 2 METHODS

**FIGURE 1:**
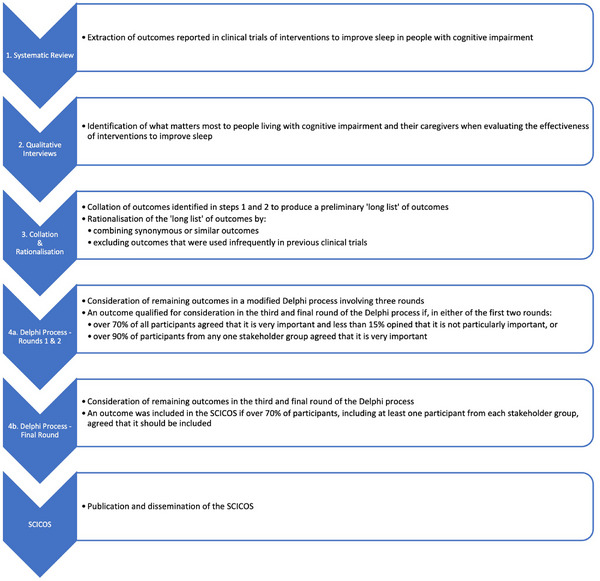
Flowchart for the SCICOS study design. SCICOS, Sleep in Cognitive Impairment Core Outcome Set.

The SCICOS protocol was published in advance^27^ and registered on the Core Outcome Measures in Effectiveness Trials (COMET) database, registration number 3250. The SCICOS was developed in accordance with the Core Outcome Set—STAndards for Development (COS‐STAD)^26^ and this report adheres to the Core Outcome Set—STAndards for Reporting (COS‐STAR).^28^ Recommendations from the CO‐research Involvement and Engagement in Dementia (COINED) model^29^ were adopted to facilitate participation of people with cognitive impairment. The SCICOS study involved a multi‐stage mixed methods process as described in the following sections (see Figure 1).

### 2.1 Systematic review

The first step in the development of the SCICOS was to conduct a systematic review to identify all the outcomes that were used in previous clinical trials of interventions to improve sleep in people with cognitive impairment. A detailed protocol for this systematic review has been published elsewhere^30^ and was prospectively registered on the International Prospective Register of Systematic Reviews (PROSPERO), registration number CRD42024556750. Briefly, Medline (Ovid), CINAHL, PsycINFO, and the Cochrane CENTRAL database were searched from inception to October 3, 2023, for controlled clinical trials of pharmacological and non‐pharmacological interventions to improve sleep in people with cognitive impairment, encompassing those with MCI and dementia. The study selection process adhered to the Preferred Reporting Items for Systematic Reviews and Meta‐Analyses (PRISMA) guidelines.^31^ Outcomes were initially extracted verbatim from included studies. Subsequently, synonymous or similar outcomes were merged by consensus of the primary project team.

### 2.2 Qualitative interviews

Qualitative semi‐structured interviews were conducted with people living with cognitive impairment and co‐morbid sleep disturbance, and/or their caregivers, to ascertain what matters most to them when determining the effectiveness of an intervention to improve sleep. In recent years, there has been growing recognition of the importance of involving people with dementia in relevant research.^32^ When developing a COS, it is considered vital to include people with lived experience of the relevant condition, as they may indicate additional outcomes of importance or emphasize different priorities compared to those suggested by clinicians or researchers.^33^ Purposive sampling was used to garner opinions from people living with varying degrees of cognitive impairment,^34^ as assessed by a consultant physician in geriatric medicine, with the severity of cognitive decline expressed according to the Reisberg Functional Assessment Staging Tool (FAST) criteria.^35^ Participants were drawn from two sources: (1) patients attending memory clinics at the Mercy University Hospital in Cork, Ireland, and (2) the Patient and Public Involvement (PPI) panels of Dementia Trials Ireland, a Clinical Trials Network funded by the Health Research Board of Ireland. Prior informed written consent was obtained from all participants. A bespoke interview topic guide was used (see protocol).^27^ Interviews were audio‐recorded and transcribed verbatim. As in similar COS development studies,^36^ the reflexive thematic analysis approach espoused by Braun and Clarke^37^ was adapted to produce a framework of outcomes deemed important to people living with cognitive impairment and their caregivers. Inductive open coding was used initially to identify outcomes. Codes with equivalent semantic meanings were merged and grouped into higher level themes. Finally, a deductive approach was taken, and codes were compared to outcomes identified by the systematic review undertaken in step 1.

### 2.3 Collation and rationalization of outcomes

Sleep outcomes identified in the systematic review (in step 1 above) were combined with additional outcomes identified by the qualitative interviews (in step 2 above), and rationalized according to the following process to produce the preliminary list of outcomes that were included in the modified Delphi process described in step 4 below:

1. As noted in the background section, a previous scoping review of the measurement of sleep in people with MCI and mild dementia revealed that many outcomes were used in only one study.^25^ As any outcome that is used so infrequently is unlikely to be considered of core importance, the following outcomes were excluded from further consideration in accordance with the previously published protocol:^27^
  - Outcomes that appeared in only one of the trials included in the systematic review mentioned in step 1 above, and
  - Outcomes that were applied to < 1% of the total number of participants involved in all trials included in the systematic review mentioned in step 1 above.
2. Outcomes that involved the total score or a sub‐score of a questionnaire were excluded.
3. The following outcomes were then added:
  - Non‐sleep outcomes identified by the systematic review described in step 1 above
  - Additional outcomes identified by the qualitative interviews described in step 2 above.
4. Remaining outcomes were rationalized by consensus among the primary project team. Similar outcomes were merged to form the initial list of outcomes that was included in the first round Delphi survey.

### 2.4 Modified Delphi process

A Delphi process is commonly used to achieve consensus when developing a COS.^38^ While multiple variations of the Delphi method have been reported in the literature, a Delphi process always involves several survey rounds in which participants are asked to score or rank a list of items, while receiving feedback on the scores of their peers in previous rounds.^39^ A modified Delphi process involving three survey rounds was used to achieve consensus regarding the outcomes that should be included in the SCICOS. While there is no agreement on the ideal sample size for a Delphi,^33^ we aimed to include sufficient lay and professional participants to ensure comprehensive representation of different perspectives at all stages of the process.^38^

#### 2.4.1 Professional participants

Internationally recognized experts in sleep and cognition were invited to participate. Experts were identified through their authorship of papers included in the systematic review mentioned in step 1 above, or through their authorship of other important and relevant scientific papers. The Delphi surveys were distributed to professional participants using Google Forms (Alphabet Inc.).

#### 2.4.2 Lay participants

Lay participants comprised people living with cognitive impairment (MCI or dementia) and co‐morbid sleep disturbance, and/or their caregivers. Lay participants were recruited from the same PPI panel of Dementia Trials Ireland and from patients attending memory clinics at the Mercy University Hospital in Cork, Ireland. Some research has suggested that the Delphi method may be unsuitable for people with dementia^40^ as their attention levels, ability to understand and retain new information, and judgment are limited by their condition.^41^, ^42^, ^43^ This may be the reason why several previous Delphis concerning different aspects of dementia care did not include people with dementia in their panels.^44^, ^45^ As detailed in the protocol,^27^ several adaptations were made to facilitate participation of people with cognitive impairment in the modified Delphi process to develop the SCICOS, including:

- Each outcome incorporated in the Delphi surveys was accompanied by an “accessible statement,” which is a simple, clear, and concise description of the outcome in plain non‐technical language.^46^
- As previous research demonstrated that Likert scales with five or more points are unsuccessful in people with dementia,^40^ a three‐point scale was used instead: “not particularly important,” “important but not critical,” and “very important.”^47^
- Paper‐based researcher‐supported Delphi surveys were used for all lay participants. A researcher‐supported survey involves a researcher supporting the participation of the person living with cognitive impairment by helping them to complete the survey.^47^

Nevertheless, perhaps due to the technical nature of many of the sleep‐related outcomes included, we found that lay participants had difficulty completing the Delphi surveys. As one participant with MCI succinctly remarked: “I think most of that (survey) was beyond the understanding of the ordinary person.” It was therefore decided to include only a representative sample of lay participants in the Delphi panel, including: two participants with MCI, two participants with mild dementia, and two caregivers for people with more advanced dementia. Similar to previous COS development studies,^48^ modifying the Delphi process in this way was deemed acceptable as the opinions of lay stakeholders had already been garnered in the qualitative interviews in step 2. Qualitative interviews are considered an effective way to obtain the perspectives of patients and caregivers,^49^ particularly as a means of informing the design of a Delphi survey for COS development,^50^ and have been used in this manner in previous COS development studies involving people with dementia.^36^, ^48^

#### 2.4.3 Delphi process and consensus criteria

The modified Delphi process consisted of three rounds of surveys in total. In the first round, participants were asked to rate the importance of each of the outcomes included in the survey before being invited to suggest any additional outcomes that they felt should be included. Additional outcomes suggested in this manner were included for consideration in the second‐round survey. Furthermore, prior to rating each outcome in the second round, participants were informed how the outcome had been rated, on average, by all participants in the first round.

In line with the Grading of Recommendations, Assessment, Development, and Evaluation (GRADE) guidelines, professional participants were asked to rate the importance of each outcome on a 9 point Likert scale.^51^ Subsequently, to align with the scores of lay participants (see section 2.4.2), scores from 1 to 3 were defined as “not particularly important,” scores from 4 to 6 were defined as “important but not critical,” and scores from 7 to 9 were defined as “very important.”^47^

An outcome qualified for consideration in the third and final Delphi survey round if, in either of the first two Delphi survey rounds:

- At least 70% of all participants regarded it as “very important” and < 15% of participants regarded it as “not particularly important,” or
- At least 90% of participants from one stakeholder group regarded it as “very important.”

In an alteration to the protocol,^27^ it was decided to disseminate a third Delphi survey round instead of hosting a final consensus meeting to facilitate ongoing participation of experts from around the world. In the third Delphi survey round, each outcome that qualified from the first two rounds was considered in turn. Participants were given a binary choice whether to include the outcome in the SCICOS. An outcome was included in the SCICOS if at least 70% of participants, including at least one participant from each stakeholder group, voted in favor of its inclusion in the SCICOS.

#### 2.4.4 Practical considerations

During the Delphi process, professional participants were also invited to give their opinion on certain ancillary matters pertaining to the practical application of the SCICOS, including the most appropriate method of measuring the included outcomes. It is important to standardize the methods of measuring outcomes in this way because otherwise, despite the development of a COS, evidence synthesis would remain impeded by incomparable scores from different measurement instruments.^52^ With respect to the outcomes included in the SCICOS, professional participants in the Delphi process were provided with a list of the measurement tools most frequently used in the clinical trials included in the systematic review mentioned in step 1 above before being asked to select their preferred option.

## 3 RESULTS

### 3.1 Systematic review

The full report of the systematic review is published elsewhere.^53^ In summary, 287 sleep outcomes were identified in 144 clinical trials involving 13,471 participants. Of these 287 outcomes, 205 were used in only one trial and were therefore excluded from further consideration in accordance with the protocol, leaving 82 sleep outcomes remaining. In addition to the aforementioned sleep outcomes, the following non‐sleep outcomes were also reported in at least 10 of the trials included in the systematic review:

- Cognition (75/144 trials)
- Behavioral disturbance (55/144 trials)
- Mood (48/144 trials)
- Functional ability (31/144 trials)
- Caregiver burden (19/144 trials)
- Quality of life (12/144 trials)
- Physical activity (10/144 trials)

### 3.2 Qualitative interviews

In all, 20 separate interviews were conducted among lay stakeholders, including:

- 9 participants with MCI, 2 of whom had a caregiver present
- 7 participants with mild dementia, 5 of whom had a caregiver present
- 2 participants with moderate dementia, both of whom had a caregiver present
- 2 caregivers of people with severe dementia

The stated aim of the interviews was to ascertain what matters most to people living with cognitive impairment and their caregivers when determining the effectiveness of an intervention to improve their sleep. The outcomes that featured most prominently in the qualitative analysis are displayed in Table 1, along with representative quotations for illustration.

**TABLE 1:** Outcomes that featured most prominently in the qualitative interviews with lay stakeholders.

Night‐time
**Total sleep time** “I crave getting more sleep” “…if you could get 4–6 hours of sleep” **Wakefulness after sleep onset** “It is the lack of consecutive sleep, the inability to get back to sleep after waking up” “that I would be able to stay asleep for the night” “a full night's sleep with no interruptions” “Even if you don't sleep too much, to get a block of uninterrupted sleep would be great” **Sleep onset latency** “I would love to go to bed and get to sleep quicker, and then stay asleep” “If I could get to sleep quicker” **Subjective sleep quality** “How you feel after the night's sleep is important” “I would like to get a better quality of sleep” “the quality of my sleep is definitely not the same as it used to be, so it would be great to improve it”

Daytime
**Daytime sleepiness & ability to function** “It would be important to avoid drowsiness” “that it wouldn't make him a zombie during the day” “I'd have more energy to get up and do things in the morning” “how you feel during the day, your energy levels, your ability to function” **Circadian rhythm & timing of sleep** “I'd like to get a more regular sleeping pattern” “I'm out of sync with society. Most people have half a day's work done by the time I'm out of bed” “If I wake up late, I wouldn't be bothered doing things” “I wake too early” “I wake very early, no matter what hour I go to bed” **Cognition** “I describe being tired as my enemy. When I'm tired, any difficulty I have increases” “It affects my concentration” “If I get a bad night's sleep, I get a brain fog” **Mood** “If you wake feeling good, you're much more inclined to feel positive” “Just improving my sleep, maybe that might improve my mood as well”

### 3.3 Collation and rationalization of outcomes

The process of collating and rationalizing the identified outcomes to produce the list of 49 outcomes that were included in the first round Delphi survey is detailed in Table 2.

**TABLE 2:** Collation and rationalization of outcomes to formulate the list of outcomes that were included in the first round Delphi survey.

Step 1	Step 2	Step 3	Step 4	Step 5
Outcomes/ outcome measures remaining following removal of those that appeared in only one trial identified by systematic review	Outcomes remaining following removal of total scores and sub‐scores of questionnaires	Outcomes remaining following removal of those used with <1% of the total participants in all trials included in the systematic review	Outcomes remaining following addition of non‐sleep outcomes from the systematic review and additional outcomes identified by qualitative interviews	Outcomes remaining following the rationalisation of outcomes to formulate first round Delphi survey
*(n=82)*	*(n=75)*	*(n=58)*	*(n=66)*	*(n=49)*
Pittsburgh Sleep Quality Index				
Epworth Sleepiness Scale				
Neuropsychiatric Inventory (sleep domain)				
Sleep Disorders inventory				
BEHAVE‐AD				
Insomnia Severity Index				
Parkinson's Disease Sleep Scale				
Number of REM bouts	Number of REM bouts			
REM Sleep Behaviour Disorder	REM Sleep Behaviour Disorder			
R‐squared (cosinor)	R‐squared (cosinor)			
AHI	AHI			
Mean oxygen saturation	Mean oxygen saturation			
Beta power	Beta power			
Delta power	Delta power			
Theta power	Theta power			
Salivary cortisol	Salivary cortisol			
TST	TST	TST	TST	TST
NTST	NTST	NTST	NTST	
DTST	DTST	DTST	DTST	
DTST/NTST	DTST/NTST	DTST/NTST	DTST/NTST	DTST/NTST
Mean duration of sleep bouts	Mean duration of sleep bouts	Mean duration of sleep bouts	Mean duration of sleep bouts	Mean duration of sleep bouts
Night mean duration of sleep bouts	Night mean duration of sleep bouts	Night mean duration of sleep bouts	Night mean duration of sleep bouts	
Peak duration of sleep bouts	Peak duration of sleep bouts	Peak duration of sleep bouts	Peak duration of sleep bouts	Peak duration of sleep bouts
Night peak duration of sleep bouts	Night peak duration of sleep bouts	Night peak duration of sleep bouts	Night peak duration of sleep bouts	
IS	IS	IS	IS	IS
IV	IV	IV	IV	IV
WASO	WASO	WASO	WASO	WASO
NNA	NNA	NNA	NNA	NNA
Number of wake bouts	Number of wake bouts	Number of wake bouts	Number of wake bouts	
Number of wakes associated with noise	Number of wakes associated with noise	Number of wakes associated with noise	Number of wakes associated with noise	
Mean duration of night‐time awakenings	Mean duration of night‐time awakenings	Mean duration of night‐time awakenings	Mean duration of night‐time awakenings	Mean duration of night‐time awakenings
NTWT	NTWT	NTWT	NTWT	TWT
DTWT	DTWT	DTWT	DTWT	
Amplitude	Amplitude	Amplitude	Amplitude	Amplitude
Acrophase	Acrophase	Acrophase	Acrophase	Acrophase
NTIB	NTIB	NTIB	NTIB	TIB
DTIB	DTIB	DTIB	DTIB	
DTIB (% observations)	DTIB (% observations)	DTIB (% observations)	DTIB (% observations)	
Sleep efficiency	Sleep efficiency	Sleep efficiency	Sleep efficiency	Sleep efficiency
Night % sleep	Night % sleep	Night % sleep	Night % sleep	
Night % awake	Night % awake	Night % awake	Night % awake	
SoL	SoL	SoL	SoL	SoL
REM latency	REM latency	REM latency	REM latency	REM latency
NREM1 latency	NREM1 latency			
NREM2 latency	NREM2 latency			
Night‐time step count	Night‐time step count	Night‐time step count	Night‐time step count	Night‐time restlessness
Night‐time activity count	Night‐time activity count	Night‐time activity count	Night‐time activity count	
Mean activity count	Mean activity count			
Arousal Index	Arousal Index	Arousal Index	Arousal Index	Arousal Index
Number of arousals	Number of arousals	Number of arousals	Number of arousals	Number of arousals
Sleep Fragmentation Index	Sleep Fragmentation Index	Sleep Fragmentation Index	Sleep Fragmentation Index	Sleep Fragmentation Index
Sleep quality	Sleep quality	Sleep quality	Sleep quality	Sleep quality
Early morning awakening	Early morning awakening	Early morning awakening	Early morning awakening	Early morning awakening
L5	L5	L5	L5	L5
L5 onset	L5 onset	L5 onset	L5 onset	L5 onset
M10	M10	M10	M10	M10
M10 onset	M10 onset	M10 onset	M10 onset	M10 onset
Relative amplitude	Relative amplitude	Relative amplitude	Relative amplitude	Relative amplitude
Number of naps	Number of naps	Number of naps	Number of naps	Number of naps
Duration of naps	Duration of naps	Duration of naps	Duration of naps	Duration of naps
Daytime % sleep	Daytime % sleep	Daytime % sleep	Daytime % sleep	Percentage time spent asleep during the day
Daytime sleep (% observations)	Daytime sleep (% observations)	Daytime sleep (% observations)	Daytime sleep (% observations)	
MESOR	MESOR	MESOR	MESOR	MESOR
Sleep onset time	Sleep onset time	Sleep onset time	Sleep onset time	Sleep onset time
Mid‐sleep time	Mid‐sleep time			
Sleep end time	Sleep end time	Sleep end time	Sleep end time	Sleep end time
Bedtime	Bedtime	Bedtime	Bedtime	Bedtime
Rising time	Rising time	Rising time	Rising time	Rising time
F‐statistic	F‐statistic	F‐statistic	F‐statistic	F‐statistic
Sleep disturbance	Sleep disturbance	Sleep disturbance	Sleep disturbance	Sleep disturbance
Use of sleep medications	Use of sleep medications	Use of sleep medications	Use of sleep medications	Use of sleep medications
Daytime dysfunction	Daytime dysfunction	Daytime dysfunction	Daytime dysfunction	Daytime dysfunction
% REM	% REM	% REM	% REM	% sleep spent in different stages
% NREM1	% NREM1	% NREM1	% NREM1	
%NREM2	%NREM2	%NREM2	%NREM2	
%NREM3	%NREM3	%NREM3	%NREM3	
%NREM4	%NREM4			
%SWS	%SWS			
REM duration	REM duration	REM duration	REM duration	Duration of different sleep stages
NREM duration	NREM duration	NREM duration	NREM duration	
NREM1 duration	NREM1 duration			
NREM2 duration	NREM2 duration			
			Measure of daytime sleepiness	Measure of daytime sleepiness
			Measure of cognition	Measure of cognition
			Measure of mood	Measure of mood
			Measure of behavioural disturbance	Measure of behavioural disturbance
			Measure of functional ability	Measure of functional ability
			Measure of daytime physical activity	Measure of daytime physical activity
			Measure of quality of life	Measure of quality of life
			Measure of caregiver burden	Measure of caregiver burden

Abbreviations: AHI, Apnoea–Hypopnoea Index; DTIB, Daytime TIB; DTST, daytime total sleep time; DTWT, daytime TWT; IS, inter‐daily stability; IV, intra‐daily variability; L5, least active five hour period; M10, most active 10 hour period; MESOR, Midline Estimated Statistic of Rhythm; NNA, number of night‐time awakenings; NTIB, night‐time TIB; NTST, night‐time total sleep time; NTWT, night‐time TWT; SoL, sleep onset latency; TIB, time in bed; TST, total sleep time; TWT, total wake time; WASO, wakefulness after sleep onset.

#### 3.3.1 Modified Delphi process

In total, there were 47 participants in the modified Delphi process. This included 41 professionals in addition to the six lay participants already described in section 2.4.2. The 41 professional participants included internationally recognized experts in sleep and cognition (See Supplementary Material in supporting information). All 47 participants completed the first‐round Delphi survey. After the first round, 13 outcomes met consensus criteria to qualify for consideration in the third and final round. Accordingly, these were not considered again in the second round. The second‐round survey included 16 additional outcomes that were suggested for consideration by respondents to the first survey. In all, 46 participants completed the second‐round survey, including a 100% response rate from professional participants. After the second round, two further outcomes met consensus criteria to qualify for consideration in the third and final round. The results of the first‐ and second‐round surveys are detailed in Table 3.

**TABLE 3:** Results of first and second round Delphi surveys. Outcomes that met consensus criteria to qualify for consideration in the third and final round are highlighted in green.

	Round one			Round two		
Outcomes	Not particularly important (%)	Important, but not critical (%)	Very important (%)	Not particularly important (%)	Important, but not critical (%)	Very important (%)
TST	10.64	8.51	80.85			
DTST/NTST	21.28	31.91	46.81	8.70	54.35	36.95
Mean duration of sleep episodes	21.28	42.55	36.17	13.04	67.39	19.57
Peak duration of sleep episodes	14.89	55.32	29.79	13.04	63.05	23.91
IS	4.26	34.04	61.70	4.35	32.61	63.04
IV	10.64	42.55	46.81	6.52	52.13	41.30
WASO	2.13	8.51	89.36			
NNA	8.51	19.15	72.34			
Mean duration of night‐time awakenings	10.64	38.30	51.06	6.52	50	43.48
TWT	17.02	38.30	44.68	10.87	54.35	34.78
Amplitude	12.76	38.30	48.94	6.52	58.70	34.78
Acrophase	19.15	42.55	38.30	13.04	65.22	43.48
TIB	14.89	31.91	53.19	4.35	45.65	50
Sleep efficiency	0	21.28	78.72			
SoL	2.13	29.79	68.08	0	15.22	84.78
REM latency	21.28	51.06	27.66	15.22	60.87	23.91
Night‐time restlessness	21.28	36.17	42.55	15.22	58.69	26.09
Arousal Index	6.38	40.43	53.19	6.52	43.48	50
Number of arousals	12.77	42.55	44.68	13.04	50	36.96
Sleep Fragmentation Index	6.38	34.04	59.58	6.52	36.96	56.52
Sleep quality	6.38	17.02	76.6			
Early morning awakening	14.89	42.55	42.55	6.52	58.70	34.78
L5	36.17	36.17	27.66	21.74	67.39	10.87
L5 onset	36.17	36.17	27.66	26.09	60.87	13.04
M10	25.53	46.81	27.66	17.39	71.74	10.87
M10 onset	34.04	40.43	25.53	26.09	63.04	10.87
Relative amplitude	27.66	42.55	29.79	19.57	60.87	19.57
Number of naps	8.51	23.40	68.09	0	36.96	63.04
Duration of naps	6.38	27.66	65.96	4.35	36.96	58.70
Percentage of time spent asleep during the day	4.25	29.79	65.96	2.17	32.61	65.22
MESOR	19.15	55.32	25.53	15.22	71.74	13.04
Sleep onset time	8.51	31.91	59.58	2.17	43.48	54.35
Sleep end time	8.51	36.17	55.32	4.35	36.96	58.70
Bedtime	8.51	38.30	53.19	6.52	43.48	50
Rising time	8.51	40.43	51.06	6.52	47.83	45.65
F‐statistic	12.77	44.68	42.55	13.04	50	36.96
Sleep disturbance	10.64	27.66	61.70	6.52	28.26	65.22
Use of sleep medication	2.13	12.77	85.10			
Day‐time dysfunction	4.26	19.15	76.59			
Percentage of sleep spent in different sleep stages	6.38	42.55	51.06	4.35	52.17	43.48
Duration of different sleep stages	6.38	48.94	44.68	6.52	54.35	39.13
Measure of daytime sleepiness	4.65	16.28	79.07			
Measure of cognition	2.13	14.89	82.98			
Measure of mood	6.38	14.89	78.72			
Measure of behavioral disturbance	6.38	25.53	68.08	2.17	30.44	67.39
Measure of functional ability	2.13	12.77	85.10			
Measure of physical activity during the day	4.65	39.53	55.81	4.88	46.34	48.78
Measure of quality of life	4.25	21.28	74.47			
Measure of caregiver burden	4.25	21.28	74.47			
NREM slow wave activity				10.87	17.39	71.74
NREM sleep spindles				10.87	34.78	54.35
Sleep‐dependent memory consolidation				13.04	47.83	39.13
Dreams/nightmares				13.04	67.39	19.57
Night‐time confusion				10.87	52.17	36.96
Screening for primary sleep disorders				2.17	28.26	69.57
Cortisol				34.78	54.35	10.87
Wake feeling refreshed				4.35	41.30	54.35
Measure of daytime fatigue/energy levels				6.52	34.78	58.70
Falls				10.87	30.43	58.70
Bodyweight				21.74	58.69	19.57
Timing of naps				6.52	67.39	26.09
Night‐to‐night variability in sleep/wake times				8.70	45.65	45.65
Time of lights out				8.70	45.65	45.65
Measure of sleep self‐efficacy				8.70	56.52	34.78
Time to institutionalization				21.74	30.43	47.83

Abbreviations: DTST, daytime total sleep time; IS, inter‐daily stability; IV, intra‐daily variability; L5, least active 5 hour period; M10, most active 10 hour period; MESOR, midline estimated statistic of rhythm; NNA, number of night‐time awakenings; NREM, non‐rapid eye movement; NTST, night‐time total sleep time; REM, rapid eye movement; SoL, sleep onset latency; TIB, time in bed; TST, total sleep time; TWT, total wake time; WASO, wakefulness after sleep onset.

After rounds one and two of the modified Delphi process, a total of 15 outcomes met consensus criteria to qualify for consideration in the third and final round. There were 44 participants in this third and final round, representing a response rate of 93.6% (comprising 95.1% of professional and 83.3% of lay participants). After the third and final round, nine outcomes met consensus criteria for inclusion in the SCICOS. These were: (1) total sleep time (TST), which measures the total duration of sleep in a defined period of time; (2) sleep onset latency (SoL), which measures the time taken to fall asleep after beginning to try to fall asleep; (3) wakefulness after sleep onset (WASO), which measures the total duration of time spent awake overnight after first falling asleep for the night; (4) number of night‐time awakenings (NNA), which measures the amount of times a person wakes up during the night; (5) sleep efficiency, which measures the percentage of total time in bed actually spent asleep; (6) sleep quality, which measures the subjective perception of the quality of sleep; (7) a measure of daytime sleepiness; (8) a measure of cognition; and (9) a measure of mood. The results of the third round are detailed in Table 4.

**TABLE 4:** Results of the third and final round Delphi survey. Outcomes that met consensus criteria for inclusion in SCICOS are shaded in green.

Outcomes	Yes, include (%)	No, exclude (%)
TST	97.73	2.27
SoL	86.36	13.64
WASO	95.45	4.55
NNA	79.55	20.45
Sleep efficiency	90.90	9.10
NREM slow wave activity	54.55	45.45
Sleep quality	90.90	9.10
Daytime dysfunction	68.18	31.82
Use of sleep medication	68.18	31.82
Measure of daytime sleepiness	90.90	9.10
Measure of cognition	86.36	13.64
Measure of mood	77.27	22.73
Measure of functional ability	63.64	36.36
Measure of quality of life	68.18	31.82
Measure of caregiver burden	59.09	40.91

Abbreviations: NNA, number of night‐time awakenings; NREM, non‐rapid eye movement; SCICOS, Sleep in Cognitive Impairment Core Outcome; SoL, sleep onset latency; TST, total sleep time; WASO, wakefulness after sleep onset.

#### 3.3.2 Practical considerations

During the modified Delphi process, professional participants were asked to provide their opinion on matters relating to the practical implementation of the SCICOS. The majority (60.98%) felt that the battery of assessments comprising the SCICOS should take between 16 and 45 minutes to administer, with 31.71% indicating that assessments should take 16 to 30 minutes and 29.27% saying they should take 31 to 45 minutes. A plurality (41.5%) recommended that sleep should be assessed over a minimum period of 7 days. Most (76.92%) agreed that TST, SoL, WASO, NNA, and sleep efficiency should be measured using both subjective and objective measurement tools. Regarding TST, the majority (75.6%) recommended that both the total night‐time sleep and the total duration of sleep over the 24‐hour day should be reported. Most (56.41%) also recommended that subjective sleep quality should be measured using a single Likert‐type question in the following manner: “How would you rate the quality of your sleep? Very good/ good/ average/ poor/ very poor.” Regarding the measurement of daytime sleepiness, while many reservations were expressed about the appropriateness of the Epworth Sleepiness Scale among this population, the majority (70.73%) recommended its use for this purpose. Most (56.1%) recommended that the Geriatric Depression Scale should be used to measure mood. Finally, opinions were quite divided regarding whether cognition should be assessed using only a short cognitive screening instrument, or a more comprehensive assessment tool, or both. A plurality (39.02%) felt that only a short cognitive screening instrument was required. Meanwhile, 26.83% felt that a more comprehensive cognitive assessment should be used, while the remaining 31.15% recommended that both be administered. A majority (65.85%) felt that the Montreal Cognitive Assessment (MoCA) is the most appropriate short cognitive screening instrument to use while, if a more comprehensive cognitive assessment is undertaken, a plurality (43.9%) recommended that the Alzheimer's Disease Assessment Scale Cognitive subscale (ADAS‐Cog) be used. The final composition of the SCICOS is set out in Table 5.

**TABLE 5 alz70890-tbl-0005:** The final Sleep in Cognitive Impairment Core Outcome Set (SCICOS).

Sleep in Cognitive Impairment Core Outcome Set (SCICOS)	
Outcomes	Practical application
Total sleep time	Both night‐time total sleep time and the total duration of sleep per 24 hour day should be measured Measure both objectively and subjectively
Sleep onset latency	Measure both objectively and subjectively
Wakefulness after sleep onset	Measure both objectively and subjectively
Number of night‐time awakenings	Measure both objectively and subjectively
Sleep efficiency	Measure both objectively and subjectively
Subjective sleep quality	Measure using a single Likert‐type question: “How would you rate the quality of your sleep over the past seven days? very good/good/average/poor/very poor.”
Measure of daytime sleepiness	Measure using the Epworth Sleepiness Scale and a single Likert‐type question: “Over the past seven days, how likely is it that you would fall asleep during the daytime without intending to, or that you would struggle to stay awake while you were doing things? no chance/slight chance/moderate chance/high chance”
Measure of mood	Geriatric Depression Scale
Measure of cognition	Montreal Cognitive Assessment +/– Alzheimer's Disease Assessment Scale‐Cognitive subscale
Sleep should be assessed over a minimum period of 7 days. Clinical trial assessments should be completed within 16 to 45 minutes.	

## 4 DISCUSSION

In recent years, there has been a marked increase in research focusing on sleep disturbances among people with cognitive impairment.^14^, ^25^ This trend reflects the rising burden of dementia^2^ and the growing preponderance of evidence implicating sleep disturbances not just in its symptomatology but also, potentially, in its pathophysiology.^11^, ^54^ A panel of sleep experts recently concluded that the three highest priorities for future research in the field of sleep and circadian rhythms in the context of aging and dementia involve developing the evidence base for interventions to improve sleep in people with, or at risk of developing, cognitive impairment.^55^ Unfortunately, most of the clinical trials that have been conducted in this field heretofore have been limited by small sample sizes.^23^, ^24^ The synthesis of data from these trials has been limited in turn by wide heterogeneity in the outcomes reported,^25^ precluding meta‐analysis^23^ and impeding the development of a robust evidence base. The SCICOS answers the previously recognized need for a COS^25^ by recommending nine core outcomes that should, insofar as practicable, be reported in all future clinical trials of interventions to improve sleep in people with MCI and dementia. These core outcome measures are: TST; SoL; WASO; NNA; sleep efficiency; subjective sleep quality; and measures of daytime sleepiness, cognition, and mood.

The SCICOS was underpinned by a comprehensive systematic review that identified all the outcomes used in previous clinical trials of both pharmacological and non‐pharmacological interventions to improve sleep in people with cognitive impairment.^53^ The involvement of lay stakeholders ensured that the SCICOS was directly informed by what matters most to people living with cognitive impairment and their caregivers. It is recognized as a limitation of the SCICOS that the relatively small sample of lay stakeholders, particularly those with more advanced dementia, may have resulted in the inadvertent oversight or omission of important outcomes. For example, while the topic of WASO frequently arose during the qualitative interviews, there was limited discussion of the behavioral disturbances (such as night‐time wandering) that may result therefrom for people with more advanced dementia. Out of practical necessity, recruitment of lay stakeholders was restricted to Ireland, which may also have introduced a bias, as the level of importance attributed to sleep outcomes may vary across different regions and cultures.^56^ Furthermore, although efforts were made to include a wide range of professional expertise from culturally diverse regions in the modified Delphi panel, the findings primarily reflect perspectives from a limited number of geographical areas, with the majority coming from Europe, North America, and Australia. Nevertheless, the Delphi panel included pre‐eminent experts in sleep and cognition from six different continents, with minimal attrition across the three rounds of the process, helping to ensure global generalizability, credibility, and relevance. It is recognized as a limitation that the Delphi process did not include a final consensus meeting. This was largely a practical solution to avoid attrition bias by facilitating the participation of experts from incompatible time zones. While a final consensus meeting is recommended,^38^ there are some other advantages to our approach, as it has been suggested that dominant voices can exert undue influence on face‐to‐face meetings.^57^ Furthermore, in previous studies, people with cognitive impairment found it difficult to attend consensus[Table alz70890-tbl-0005] meetings.^48^

Sleep is complex and multi‐dimensional, and is thus measurable across many different levels and aspects.^58^ Furthermore, in addition to measuring sleep per se, it is important to consider the many ways in which sleep affects other aspects of health and well‐being.^59^, ^60^ Given this complexity, it is recognized that the suitability of outcomes may vary widely depending on the context and objectives of a particular clinical trial. For example, efficacy studies (usually involving small, well‐defined samples) and effectiveness studies (usually involving large community samples in routine practice settings) may require different outcomes.^61^ Rather than being prescriptive, therefore, the SCICOS recommends a core set of outcomes that should be reported insofar as practicable.

The outcomes included in the SCICOS arguably encompass the most important aspects of sleep, thereby providing a meaningful overview of sleep‐related health. It should be possible to complete the SCICOS assessments within the recommended 16 to 45 minute timeframe, which is important because it is recognized that the participation of people with dementia in research is affected by the time commitment involved.^62^ All outcomes that were identified as being of paramount importance by lay stakeholders in the qualitative interviews are included in the SCICOS, except for outcomes relating to circadian rhythm and the timing of sleep. This is recognized as a limitation of the SCICOS, and future clinical trials should consider including outcomes relating to circadian rhythm and the timing of sleep, if appropriate.

The SCICOS insures against the well‐recognized discrepancy between subjective and objective measures of sleep^63^ by recommending that both be measured, when possible. Indeed, this will provide further opportunities to explore the reasons for the discrepancy. The subjective self‐perception of sleep quality and duration is undoubtedly prone to recall bias, particularly among people with cognitive impairment.^64^, ^65^ It may, therefore, be preferable to rely on objective measures or on observer/informant reports, particularly in relation to people with more advanced cognitive impairment. While proxy caregiver reports are also prone to sleep misperception,^20^ systematic behavioral observations of sleep undertaken by trained researchers in institutional settings^66^ have demonstrated excellent inter‐rater reliability in previous clinical trials.^67^ Studies demonstrating the discrepancy between subjective and objective measures of sleep in older people have suggested that both are important as they measure different aspects of sleep.^68^ Healthy sleep is multi‐dimensional,^58^ and the subjective perception of sleep may convey unique information about a person's well‐being relative to objective measures.^69^ The SCICOS is not prescriptive regarding the devices that should be used to obtain the objective sleep measures. There are many available methods of measuring sleep objectively, each with its own associated advantages and disadvantages^70^ and varying degrees of accuracy compared to gold standard polysomnography^71^. Indeed, the use of polysomnography itself has inherent limitations in people with dementia.^64^, ^72^ A panel of international sleep experts has expressed confidence in actigraphy/accelerometry devices as long as they are of “research quality,” having obtained the relevant regulatory approvals, such as the medical device Conformité Européene (CE) mark and Food and Drug Administration (FDA) approval.^55^ While it has previously been suggested that a 14 day period of measurement is ideal,^73^ the SCICOS recommends that sleep be measured over a minimum period of 7 days, which should be sufficient to provide reliable estimates of sleep parameters in people with cognitive impairment.^74^

To help ensure comparability of data from future clinical trials, which is the primary objective of any COS, the SCICOS also makes recommendations regarding the tools that should be used to measure subjective sleep quality, daytime sleepiness, cognition, and mood. With respect to each of the latter three outcomes, professional participants in the Delphi process were asked to select their preferred measurement tools from a list compiled from those most frequently used in the clinical trials that were included in the systematic review. It is recognized as a limitation of the study that this methodology may have resulted in other, more appropriate, measurement tools being omitted from consideration. The majority recommended that subjective sleep quality should be measured using a single Likert‐type question, a widely recognized and commonly used method of measuring this metric.^75^ While a majority ultimately recommended the Epworth Sleepiness Scale to measure daytime sleepiness, there were many reservations expressed regarding its suitability for people with cognitive impairment. Several participants opined that, in the absence of a more appropriate tool, it may be useful to add a single Likert‐type question to assess sleepiness and, if possible, obtain corroborative information from a caregiver.

In conclusion, informed by solicited knowledge of what matters most to people with cognitive impairment and their caregivers, who have lived experience of the consequences of sleep disturbances, the SCICOS used a panel of pre‐eminent international experts to produce, by consensus, a COS consisting of nine outcomes for future clinical trials of interventions to improve sleep in people with MCI and dementia. By recommending a standard minimum set of outcomes and recommending standardized methods by which these should be measured, the SCICOS will help ensure that future clinical trials produce more coherent and reliable data to inform clinical practice.

## CONFLICT OF INTEREST STATEMENT

The authors declare no conflicts of interest. All author disclosures are available in the supporting information.

## CONSENT STATEMENT

This study was carried out in accordance with the Declaration of Helsinki. Prior informed consent to participation was obtained from all participants. Ethical approval for the study was granted by the clinical research ethics committee (CREC) of the Cork Teaching Hospitals, reference numbers: ECM 4 (c) 06/02/2024 & ECM 5 (7) 20/02/2024 & ECM 4 (dd) 30/07/2024.

## Supporting information



Supporting Information

Supporting Information
